# Retraction Note: Comparison of minimally invasive Ivor Lewis esophagectomy and left transthoracic esophagectomy in esophageal squamous cell carcinoma patients: a propensity score-matched analysis

**DOI:** 10.1186/s12885-022-09471-x

**Published:** 2022-04-14

**Authors:** Qi Wang, Zixiang Wu, Tianwei Zhan, Shuai Fang, Sai Zhang, Gang Shen, Ming Wu

**Affiliations:** Department of Thoracic Surgery, 2nd Affiliated Hospital, School of Medicine, Zhejiang University, 88 JieFang Rd, Hangzhou, 310009 China

**Retraction Note: BMC Cancer 19, 500 (2019)**

**https://doi.org/10.1186/s12885-019-5656-7**

The Editor has retracted this article because an investigation by Zheijang University Academic Committee concluded that some of the surgical cases reported were the result of the sharing of clinical data and the ethics approval provided for this study does not cover all the cases included.

None of the authors has responded to correspondence from the Editor about this retraction.

